# Production of high-concentration *n*-caproic acid from lactate through fermentation using a newly isolated *Ruminococcaceae* bacterium CPB6

**DOI:** 10.1186/s13068-017-0788-y

**Published:** 2017-04-21

**Authors:** Xiaoyu Zhu, Yan Zhou, Yi Wang, Tingting Wu, Xiangzhen Li, Daping Li, Yong Tao

**Affiliations:** 1 Key Laboratory of Environmental and Applied Microbiology, Chinese Academy of Sciences, Environmental Microbiology Key Laboratory of Sichuan Province, Chengdu Institute of Biology, Chinese Academy of Sciences, Chengdu, 610041 People’s Republic of China; 2 Department of Biosystems Engineering, Auburn University, Auburn, AL 36849 USA; 3 College of Life Science, Sichuan University, Chengdu, 610041 People’s Republic of China

**Keywords:** Caproic acid, Hexanoate, Lactate, *Ruminococcaceae* bacterium, Organic waste, Chain elongation

## Abstract

**Background:**

*n*-Caproic acid (CA), as a medium-chain carboxylic acid, is a valuable chemical feedstock for various industrial applications. The fermentative production of CA from renewable carbon sources has attracted a lot of attentions. Lactate is a significant intermediate waste in the anaerobic breakdown of carbohydrates that comprise 18–70% of the chemical oxygen demand (COD) in municipal and some industrial wastewaters. Recently, researchers (including our own group) reported the CA production using lactate as electron donor with newly identified microbiome systems. However, within such processes, it was hard to determine whether the CA production was completed by a single strain or by the co-metabolism of different microorganisms.

**Results:**

Here, we report the CA production using lactate as electron donor using the strain CPB6, which we isolated from a microbiome for CA production as described previously. Strain CPB6 is affiliated with *Clostridium* cluster IV of the family of *Ruminococcaceae* based on 16S rRNA gene sequence analysis. The strain prefers acidic initial pH condition (pH 5.0–6.5), and the temperature ranging from 30 to 40 °C for CA production. In a fed-batch fermentation with non-sterilized lactate-containing organic wastewater as feedstock, strain CPB6 produced 16.6 g/L CA (from 45.1 g/L lactate) with a maximum productivity of 5.29 g/L/day. Enzyme assays with crude cell extract showed that CPB6 can metabolize acetate and butyryl-CoA to produce *n*-butyric acid, and acetate/*n*-butyrate and caproyl-CoA to produce CA, respectively.

**Conclusion:**

This study demonstrated that high concentration of CA production can be obtained by a newly isolated pure culture CPB6. This strain can be employed as a powerful workhorse for high-efficient CA recovery from lactate-containing waste streams. Our preliminary investigation suggested that the CA production from lactate in strain CPB6 might be via the chain elongation pathway of the reverse β-oxidation; the detailed mechanism, however, warrants further investigation using various molecular microbiology techniques.

**Electronic supplementary material:**

The online version of this article (doi:10.1186/s13068-017-0788-y) contains supplementary material, which is available to authorized users.

## Background

Medium-chain carboxylic acid, *n*-caproic acid (CA), is a valuable platform chemical for various industrial applications, including as a chemical precursor for producing flavor compounds and aviation fuels [1, 2]. In addition, CA has the potential to be used as a natural antimicrobial agent [3]. Up to now, the primary mechanism for CA formation in the fermentation is through the chain elongation (reverse β oxidation), in which functional microbes elongate acetate/*n*-butyrate (butyrate hereafter, unless otherwise indicated) with two carbons (acetyl-CoA, being derived from ethanol) each time, to convert them into chemicals with six or above carbons, in sequence [4, 5]. Due to the low solubility and thus easy separation, the recovery of CA from organic wastes has been emerged as a popular strategy for organic waste treatment, where macromolecules are firstly decomposed into short-chain carboxylates, e.g., acetate and butyrate, which are then converted into CA with ethanol supplemented as electron donor [6–10].

Lactate is an important intermediate in the anaerobic breakdown of carbohydrates that comprise ~18% of the chemical oxygen demand (COD) in municipal wastewaters and up to ~70% of the COD in some food processing wastewaters [11–14]. The conversion of lactate to CA had been described in pure culture studies using *Megasphaera elsdenii* [15] and reactor systems [16, 17]; however, the CA production from lactate was not suitable to be considered as an effective and worthwhile approach for organic waste treatment, because the CA produced was only the negligible byproduct with low titer (<1.0 g/L) beside propionate or butyrate as the main product. Recently, we reported a unique microbiome that is predominated by *Clostridium* cluster IV, which can efficiently convert lactate into high level of CA as the primary product [18]. In batch fermentation with sufficient lactate addition, the maximum CA titer reached up to 23.4 g/L, with a CA productivity of 2.94 g/L/day. Soon afterwards, Kucek et al. [14] developed a continuous process to produce CA from lactate using a microbiome dominated by *Acinetobacter* spp. At a hydraulic retention time (HRT) of 1.5 days, a CA productivity of 3.03 g/L/day was reached. These results together demonstrated that besides ethanol, lactate, which is often present in various organic wastes, can also be used as an electron donor and carbon source for high production of CA.

Up to now, *M. elsdenii* is the only pure culture known to be capable of converting lactate into CA [15, 19]. However, in none of the above cases for CA production from endogenous lactate [14, 18, 20], *M. elsdenii* was detected. So, *M. elsdenii* was not likely the main strain that is responsible for the lactate-to-CA conversion in these processes. In order to gain a further insight into these processes and achieve a further development of this new technology, it is desirable to isolate and metabolically characterize the key microorganism contributing to these processes from these reactor microbiome systems.

In this study, we isolated a bacterial strain CPB6 (belongs to *Clostridium* cluster IV of the family *Ruminococcaceae*) from the reactor microbiome capable of producing high concentration of CA from lactate [18]. We investigated the effects of various operational parameters on the CA production efficiency by CPB6. We further demonstrated CA production using this strain from real wastewater containing high concentration of lactate under non-sterilized and sterilized conditions. Finally, the metabolic mechanism of this new process was also explored preliminarily.

## Results and discussion

### Culture morphology and phylogeny

After a series of continuous purification, a lactate-utilizing and CA-producing strain CPB6 was isolated from the fed-batch reactor microbiome described previously [18]. After a 3-day incubation on the agar plate at 30 °C, the colonies of the anaerobic bacterium strain CPB6 displayed white circular smooth colonies with a diameter of 1–2 mm. In liquid culture, microscopic analysis revealed that strain CPB6 was short, rod-shaped, spore-forming bacteria of 1.5–3.0 μm length and approximately 0.5 μm width (Additional files 1, 2: Figure S1).

**Fig. 1:**
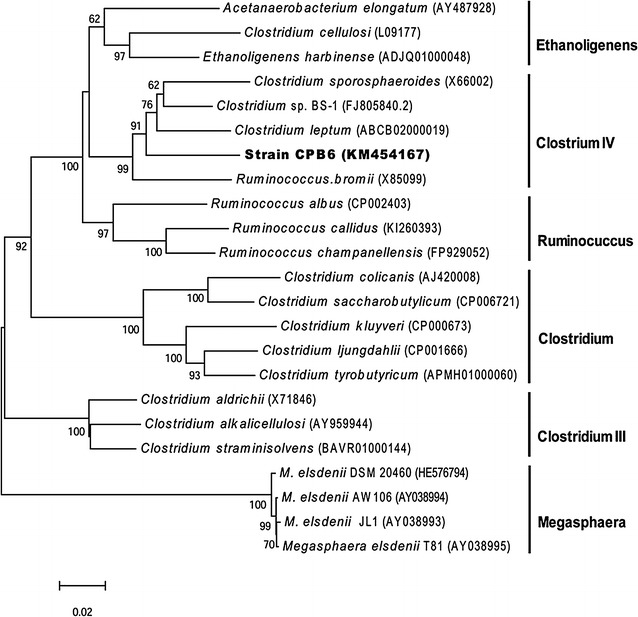
Neighbor-joining phylogenetic tree based on 16S rRNA gene sequences. The *numbers at the nodes* indicate the level of bootstrap values (1000 replications, >50%). *Bar* 0.02 indicates substitutions per nucleotide position

We sequenced the 16S rRNA gene of the strain (GenBank accession No. KM454167) (Additional file 1). Based on the sequence analysis, the CPB6 strain was affiliated with *Clostridium* cluster IV of the family of *Ruminococcaceae* (Fig. 1). The strain generally showed low sequence similarity with the existing strains whose 16S rRNA sequences are included in GenBank. When compared to the type species in the GenBank, it was closest to *Clostridium leptum* and *Clostridium sporosphaeroides* with 92.6 and 91.7% similarity of 16S rRNA sequence, respectively. While compared to the well-known CA-producing bacteria, it showed generally low similarity with *Clostridium kluyveri* (82.4%) and *Megasphaera elsdenii* (75.7%), but higher similarity with *Clostridium* sp. BS-1 (93.6%). The phylogenetic analysis implicated that CPB6 might belong to a new clade (genus) of the family *Ruminococcaceae*.

### Strain CPB6 can utilize lactate to produce CA as the main product

**Fig. 2:**
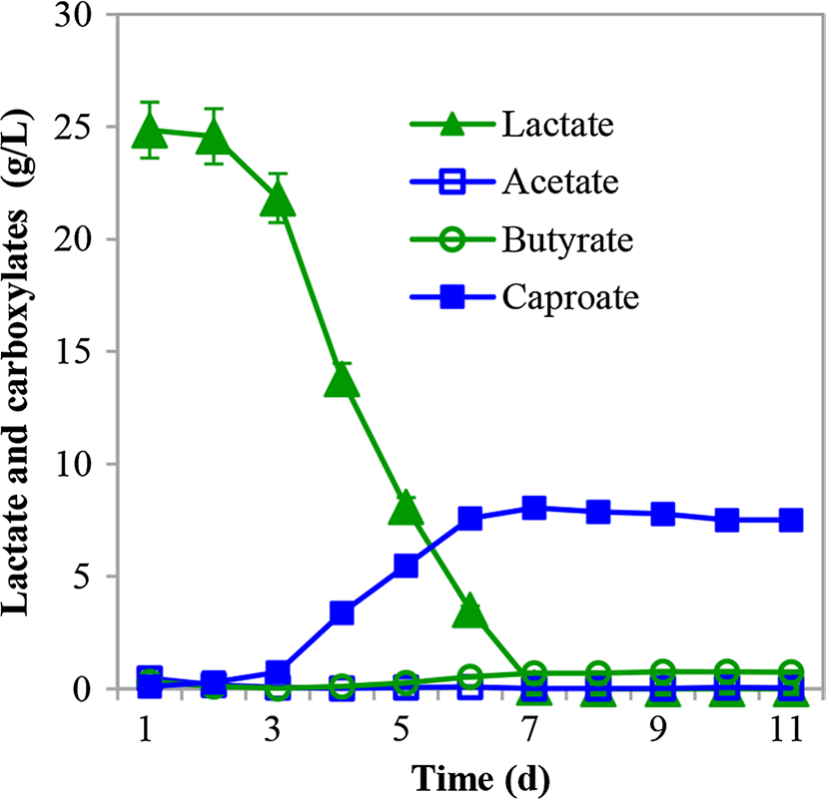
Caproic acid (CA) formation from lactate by strain CPB6 in a fed-batch fermentation

As shown in Fig. 2, the concentration of CA increased in parallel with the continuous consumption of d, l-lactate. Then CA concentration reached the plateau after the exhaustion of lactate on the 6th day of the fermentation, with 8.07 g/L of CA generated from 24.85 g/L of lactate. From the 4th day of the fermentation, butyrate started to accumulate and reached approximately 0.75 g/L by the end of the fermentation. No propionate was detected while only negligible acetate was generated during the fermentation. These results indicated that the strain CPB6 can utilize d, l-lactate to produce CA as the main product.

Other lactate-utilizing bacteria reported in previous studies generally fermented lactate to short-chain fatty acids (SCFAs), e.g., butyrate and propionate [21, 22]; few can synthesize medium-chain fatty acids (MCFAs), e.g., CA [15]. For example, *Clostridium leptum* is the type strain of the closest relative species to strain CPB6. It is also a lactate utilizer, but it only produces butyrate, rather than CA, from lactate [23]. *Megasphaera elsdenii* is the only type strain that can produce CA from lactate, but in this case, CA is only a byproduct alongside the main metabolites including butyrate, propionate, and valerate [15, 24, 25]. The final CA titers were usually very low from the fermentation with either the pure culture of *M. elsdenii* or with a microbiome part of which was composed of *M. elsdenii* [17]. Here, the final CA concentration from the fermentation with strain CPB6 is >30 times higher than that was reported with *M. elsdenii* (8.07 versus 0.23 g/L [15]). Therefore, strain CPB6 is concluded as a novel key isolate that can efficiently convert lactate to CA as dominated product.

### Effect of initial pH on CA production from lactate

**Fig. 3:**
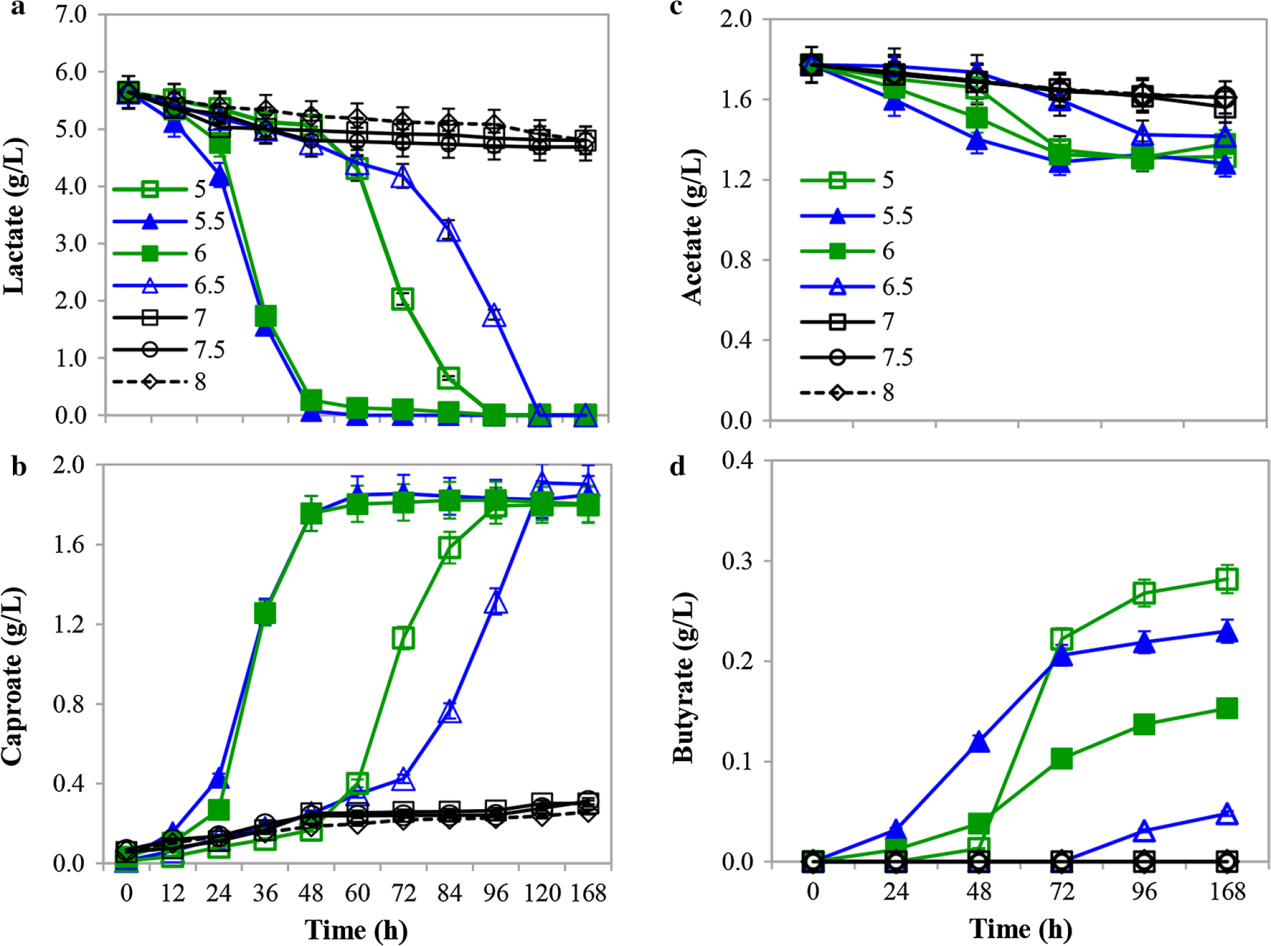
Effect of pH on caproic acid (CA) production from lactate: lactate consumption (**a**), CA production (**b**), acetate consumption (**c**), and butyrate accumulation (**d**)

The pH of fermentation is a crucial factor influencing the CA production by affecting the concentration of the undissociated CA. It was reported that, at pH 5.5, when the concentration of the undissociated CA exceeds 0.79 g/L (7 mM), it will significantly inhibit the activity of microbial cells [10]. The effect of pH on CA production by strain CPB6 was illustrated in Fig. 3 and Additional file 2: Figure S2, respectively. When the initial pH of the fermentation ranged from 5.0 to 6.5, about 1.80 g/L CA was produced as the primary end product. No obvious difference was observed in terms of the final CA titer and production rate (Fig. 3b). In addition, the range of initial pH from 5.0 to 6.5 was also very favorable for CPB6 cell growth (Additional file 2: Figure S2A), which was an important reason for high CA production at this pH range. However, as the initial pH of fermentation was adjusted to 7.0, 7.5, or 8.0, less lactate (0.80, 0.96, and 0.95 g/L, respectively) was consumed with little amount of CA (0.32, 0.25, and 0.17 g/L, respectively) being produced (Fig. 3c). It was observed that the strain experienced a long lag phase about 2 days, and reached a much lower maximum cell density (Additional file 2: Figure S2A). The CA production rate at initial pH conditions of 5.0–6.5 was almost the same, but it decreased to much lower levels when the initial pH was increased to 7.0–8.0 (Additional file 2: Figure S2C). In addition, the accumulation of butyrate, an undesirable byproduct decreasing the yield of CA, was decreased from 0.28 to 0.05 g/L with the initial pH increased from 5.0 to 6.5, as shown in Fig. 3d. Trace amounts of butyrate were produced when the initial pH was higher than 7.0.

In contrast to strain CPB6, other CA-producing bacteria exhibit higher CA titers under neutral conditions [6, 26, 27]. A mixed culture dominated by *C. kluyveri* produced much more CA at pH 7.0 than at pH 5.5 [6]. The lactate-utilizing CA-producing strain *M*. *elsdenii* also prefers neutral conditions [15]. *M. elsdenii* produces more CA when pH is maintained at 7.0 than when pH is uncontrolled with glucose as the substrate [27]. Choi et al. [28] demonstrated that *M*. *elsdenii* prefers neutral conditions to acidic conditions with sucrose as the substrate. *Clostridium* BS-1, a member of *Clostridium* cluster IV, also requires neutral pH to convert d-galactitol to CA [29]. However, in organic waste fermentation, some studies of CA production by reactor microbiomes have chosen to control the pH at 5.5 or even lower to eliminate competition from acetoclastic methanogens, which exhibit greater growth under neutral conditions, or to regulate the metabolic pathway toward chain elongation rather than the acrylate pathway [10, 14]. The efficiency of CA production in mixed culture is negatively affected by the lower pH control strategy compared to that of a pure culture for two reasons: (1) the decreased activity of CA producers; (2) the increased concentration of toxic undissociated CA, particularly without in-line extraction [1, 10, 14]. However, strain CPB6, the dominant bacterium in CA-producing mixed cultures, might represent a smart solution to eliminating these two problems due to its initial acid preference and the significant increase in pH during CA production (Additional file 2: Figure S3), circumventing competition from other bacteria, such as acetoclastic methanogens and the toxicity of undissociated CA as CA accumulates.

### Effect of temperature on CA production from lactate

**Fig. 4:**
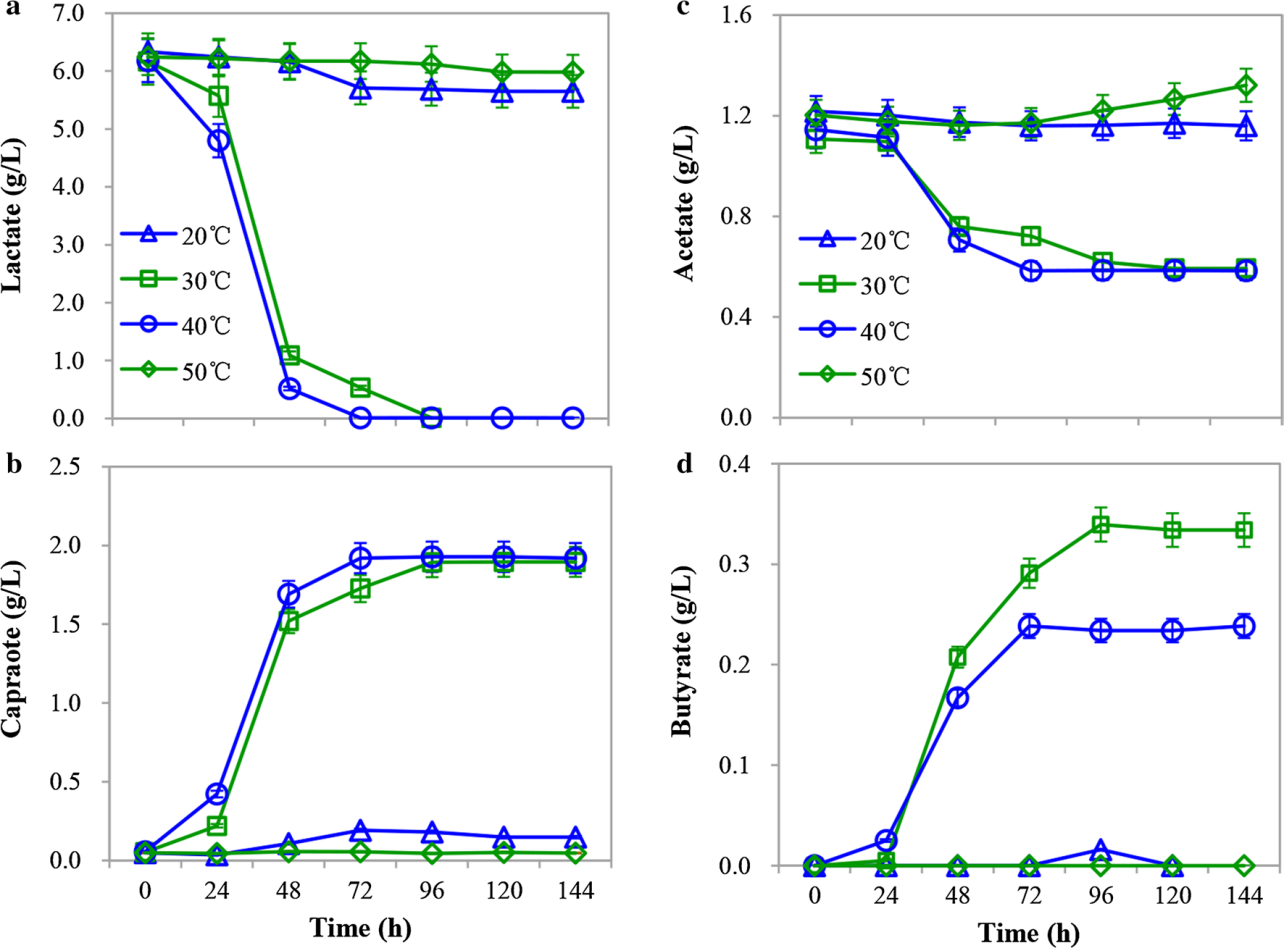
Effect of temperature on caproic acid (CA) production from lactate: lactate consumption (**a**), CA production (**b**), acetate consumption (**c**), and butyrate accumulation (**d**)

The effect of temperature on lactate fermentation by strain CPB6 was investigated under 20, 30, 40, and 50 °C. Strain CPB6 could grow at 20 °C, but very little CA (0.14 g/L) was produced. By contrast, 1.84 and 1.86 g/L of CA were obtained at 30 and 40 °C, respectively (Fig. 4b), which was well in accordance with the good cell growth under these conditions (Additional file 2: Figure S2B). Correspondingly, the CA production rate was found to be pretty similar for 40 °C and 30 °C (Additional file 2: Figure S2D). Meanwhile, butyrate production at 30 °C was slightly higher than that at 40 °C (Fig. 4d). Only slight cell growth was observed when the temperature was increased to 50 °C, resulting in negligible CA and butyrate production (Fig. 4; Additional file 2: Figure S2B). The tested temperature response of the strain CPB6 clearly indicated that it prefers the temperature ranging from 30 to 40 °C for growth and CA production. Similarly, most other CA producers are mesophilic, e.g., *C. kluyveri* [30] and *M. elsdenii* [15], as reported previously.

### High level of CA production from lactate-containing wastewater

**Fig. 5:**
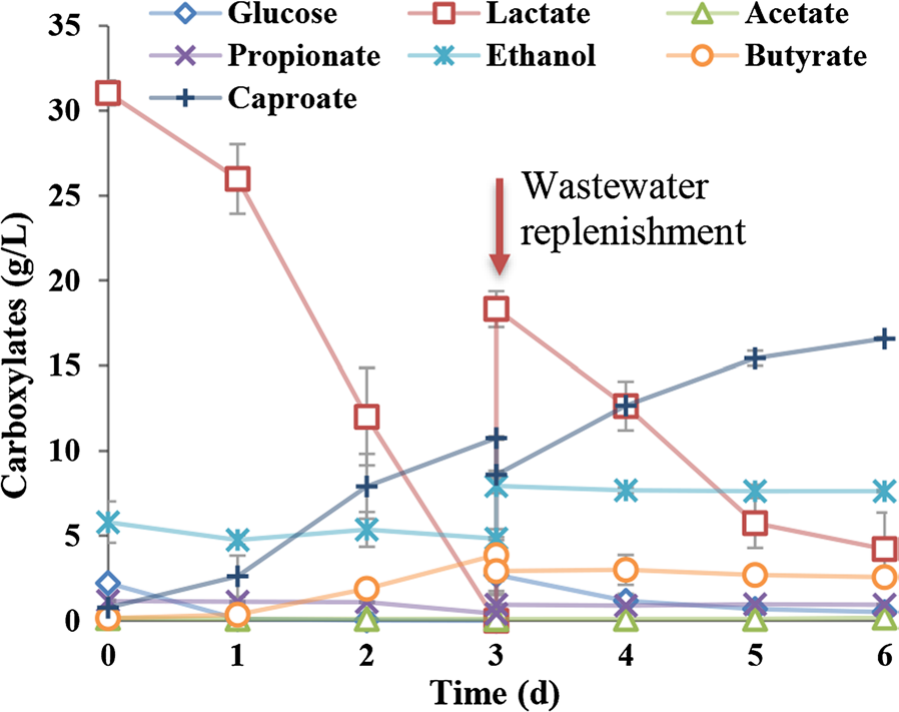
Caproic acid (CA) recovery from lactate-containing wastewater by strain CPB6 under non-sterilized conditions. The fermentation was started using the mixed wastewater as described in the “Methods” section. When the wastewater was depleted, additional wastewater was added to support the further CA production

Further experiment was conducted to investigate whether the strain CPB6 can be employed to recover CA from real wastewater. As shown in Fig. 5, with an initial fermentation cycle and a further wastewater replenishment, a final CA concentration of 16.6 g/L was obtained with rapid consumption of lactate in the wastewater. The maximum CA productivity was 5.29 g/L/day, which was nearly two times higher than the results from our previous report when mixed culture was used [18]. The control reactor with sterile wastewater showed a production of CA of 15.9 g/L with a maximum productivity of 5.63 g/L/day (Additional file 2: Figure S4). These results indicated that CA can be recovered from waste streams with the pure culture of strain CPB6 under non-sterilized conditions. Furthermore, no significant propionate or butyrate accumulation was observed (Fig. 5), which is different from other well-known CA producers. This also explains why a high CA yield from lactate can be obtained with strain CPB6.

### Effects of acetate and butyrate supplementation on CA production from lactate

In the production of CA via chain elongation (reverse β oxidation) in many microorganisms as discussed above, acetate can serve as the starting substrate, while butyrate can be either the starting substrate or as an intermediate product (Table 1). In strain CPB6, butyrate production was also detected when lactate was used as the electron donor for CA production. So, here we tested the effects of acetate and butyrate supplementation on CA production in strain CPB6.

**Table 1:** Possible pathways from literature and the calculation of the Gibbs free energy changes for caproic acid formation

No.	Pathways		References
Equation (1)	3Ethanol + 4H_2_ + 2H^+^→Caproate + 4H_2_O	$\Delta G_{r}^{0\prime }$ = − 96.71 kJ/mol	[31]
Equation (2)	Butyrate + Ethanol + 2H_2_ + H^+^→ Caproate + 2H_2_O	$\Delta G_{r}^{0\prime }$ = − 48.41 kJ/mol	
Equation (3)	Butyrate + 2CO_2_ + 6H_2_ → Caproate + 4H_2_O	$\Delta G_{r}^{0\prime }$ = − 143.29 kJ/mol	
Equation (4)	Ethanol + H_2_O → Acetate + 2H_2_ + H^+^	$\Delta G_{r}^{0\prime }$ = 9.65 kJ/mol	[5]
Equation (5)	4Ethanol + 4Acetate → 4Butyrate + 4H_2_O	$\Delta G_{r}^{0\prime }$ = − 38.65 kJ/mol	
Equation (6)	Ethanol + Butyrate → Caproate + H_2_O	$\Delta G_{r}^{0\prime }$ = − 38.76 kJ/mol	
Equation (7)	Lactate + H_2_O → Acetate + 2H_2_ + CO_2_	$\Delta G_{r}^{0\prime }$ = − 6.9 kJ/mol	[18]
Equation (8)	Lactate + Acetate + H^+^ → Butyrate + H_2_O + CO_2_	$\Delta G_{r}^{0\prime }$ = − 97.5 kJ/mol	
Equation (9)	Lactate + Butyrate + H^+^ → Caproate + H_2_O + CO_2_	$\Delta G_{r}^{0\prime }$ = − 80.9 kJ/mol	

**Fig. 6:**
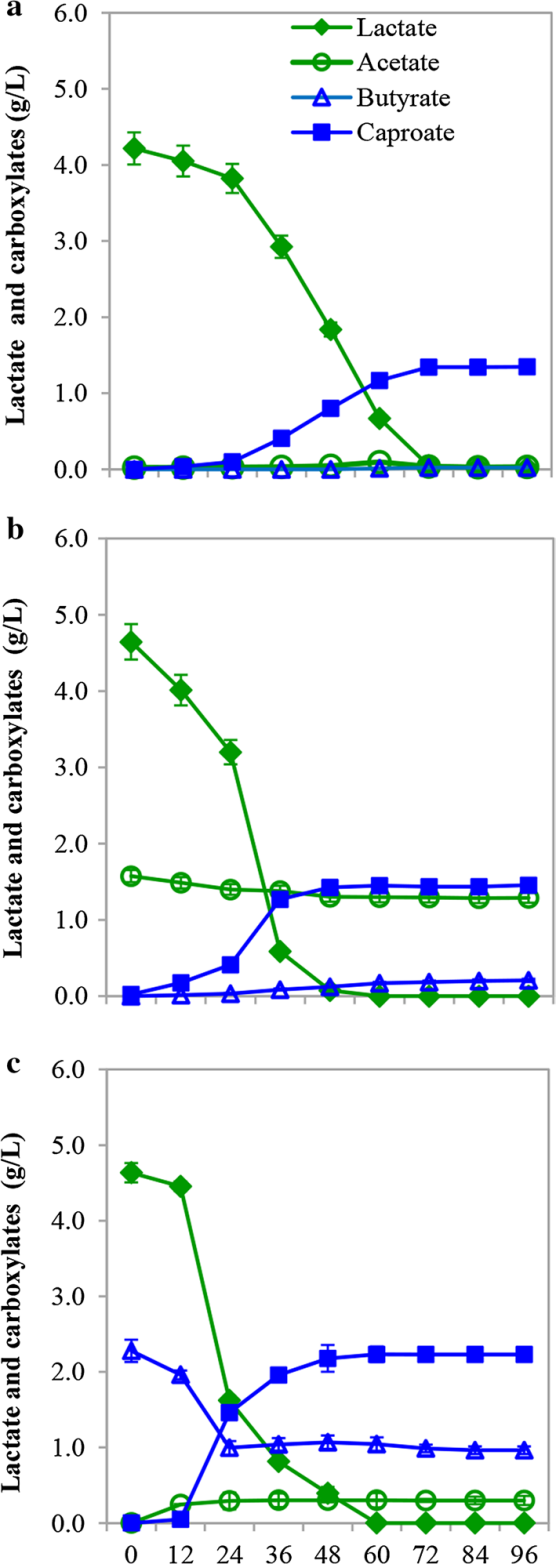
Caproic acid (CA) production in strain CPB6 using different substrates: **a** lactate; **b** lactate and acetate; **c** lactate and butyrate

As shown in Fig. 6, when either acetate or butyrate (besides lactate) was supplemented to the fermentation, strain CPB6 simultaneously used lactate and acetate or lactate and butyrate from the very beginning. Compared with the control reaction (only lactate is provided), the lag phase for the fermentation with acetate or butyrate supplementation was shortened from 24 to 12 h, indicating that acetate and butyrate can accelerate CA production significantly. Based on a carbon balance calculation for the fermentation, it was found that in the presence of acetate or butyrate, the lactate requirement for one mol of CA production reduced from 4.29 mol to 3.83 or 2.68 mol, respectively (Table 2). Meanwhile, the production of hydrogen decreased from 2.75 mol to 2.36 or 1.85 mol, respectively. These results demonstrated that the supplemented acetate and butyrate might have been directly involved in the CA synthesis. In addition, it has been described that lactate can be converted to butyrate via chain elongation [2]. Therefore, here we preliminarily deduced that the CA production from lactate in strain CPB6 might be also via chain elongation pathway. In addition, we also conducted experiments with only acetate or butyrate as substrate without lactate added. The results demonstrated that no caproate was produced (Additional file 2: Figure S5). This indicated that acetate or butyrate can be supplemented to lactate for CA production in the strain CPB6, but itself (acetate or butyrate) cannot serve as the sole carbon source for this microorganism.

**Table 2:** Stoichiometric balances for the fermentation using strain CPB6 grown on different substrates

	1 mol caproate produced from		
	Lactate	Lactate, acetate	Lactate, butyrate
Lactate	−4.29^a^	−3.83^a^	−2.68^a^
Acetate	0.02	−0.43^a^	0.26
Butyrate	0.03	0.17	−0.82^a^
Caproate	1.00	1.00	1.00
Hydrogen	2.75	2.36	1.85

Unit is moles

^a^ “−” Means consumption

### Key reactions and enzymes for CA production

There is no previous reference demonstrating that lactate can be converted into CA through the chain elongation pathway. In the chain elongation pathway in *C. kluyveri*, acetoacetyl-CoA, 3-hydroxybutyryl-CoA, crotonyl-CoA, and butyryl-CoA are intermediates for butyrate formation [5], while 3-keto-caproyl-CoA, 3-hydroxyhexanoyl-CoA, hex-2-enoyl-CoA, and caproyl-CoA are intermediates for CA production [2, 32]. According to the literature [2, 33], there are two possible reactions for CA production (which were actually inferred from reactions for butyrate production): (1) caproyl-CoA + acetate → caproate + acetyl-CoA; (2) caproyl-CoA + butyrate → caproate + butyryl-CoA. Here, we thus further carried out enzyme assays to investigate whether the last key reactions for CA production exist in strain CPB6 (Table 3). Our results demonstrated that, in the presence of excessive caproyl-CoA, 1.15 mM acetate led to 1.14 mM of CA production, and the utilization of 0.77 mM butyrate resulted in 0.76 mM of CA formation. While in the control test, the concentration of acetate (3.89 mM) or butyrate (4.02 mM) remained constant all the time and no CA production was detected. This experiment provided the evidence that both key reactions as described above exist in strain CPB6, implying that both acetate and butyrate are involved in the last step of CA formation. However, the activity of the enzyme (caproyl-CoA: acetate CoA transferase) responsible for Reaction (1) was higher than that of the enzyme (caproyl-CoA: butyrate CoA transferase) for Reaction (2) (Table 4). Likewise, with the presence of excessive butyryl-CoA, 0.45 mM acetate was consumed leading to about almost equal amount of butyrate (0.42 mM) production (Table 3), while the control test showed no butyrate production. It further indicated the existence of the enzyme butyryl-CoA: acetate CoA transferase in strain CPB6. In addition, our experiments illustrated that the CA production rates were 3.5 and 2.3 times higher than that of the butyrate production (Table 4), respectively, which could well explain why CA was always the predominant product in the fermentation with strain CPB6.

**Table 3:** Key possible reactions for caproic acid (CA) formation and stoichiometric fermentation balances using the crude cell extract of strain CPB6 grown on different substrates

	Acetate^a^	Butyrate^a^	Caproate^a^
Caproate *formation*			
Rec. (1) Caproyl-CoA + Acetate → Caproate + Acetyl-CoA	−1.15^b^	nd^c^	1.14
Rec. (2) Caproyl-CoA + Butyrate → Caproate + Butyryl-CoA	nd^c^	−0.77^c^	0.76
*Butyrate formation*			
Rec. (3) Butyryl-CoA + Acetate → Butyrate + Acetyl-CoA	−0.45^c^	0.44^c^	nd^c^

Supplement of substrates (acetate, butyrate, butyryl-CoA, and caproyl-CoA) were sufficient

Control measurements (without crude enzyme solution) were not shown for no butyrate or caproate was produced

^a^ The unit is mM

^b^ “−” Substrate consumption

^c^ Not detected

**Table 4:** Comparison of the specific production rates of the three key reactions for chain elongation in strain CPB6

	R1^a^	R2^a^	R3^a^	R1/R3	R2/R3
Strain CPB6	0.162	0.108	0.046	3.522	2.348

R1: Caproyl-CoA + Acetate → Caproate + Acetyl-CoA; R2: Caproyl-CoA + Butyrate → Caproate + Butyryl-CoA; R3: Butyryl-CoA + Acetate → Butyrate + Acetyl-CoA

^a^ The unit of rates is mg product/g biomass/min

**Fig. 7:**
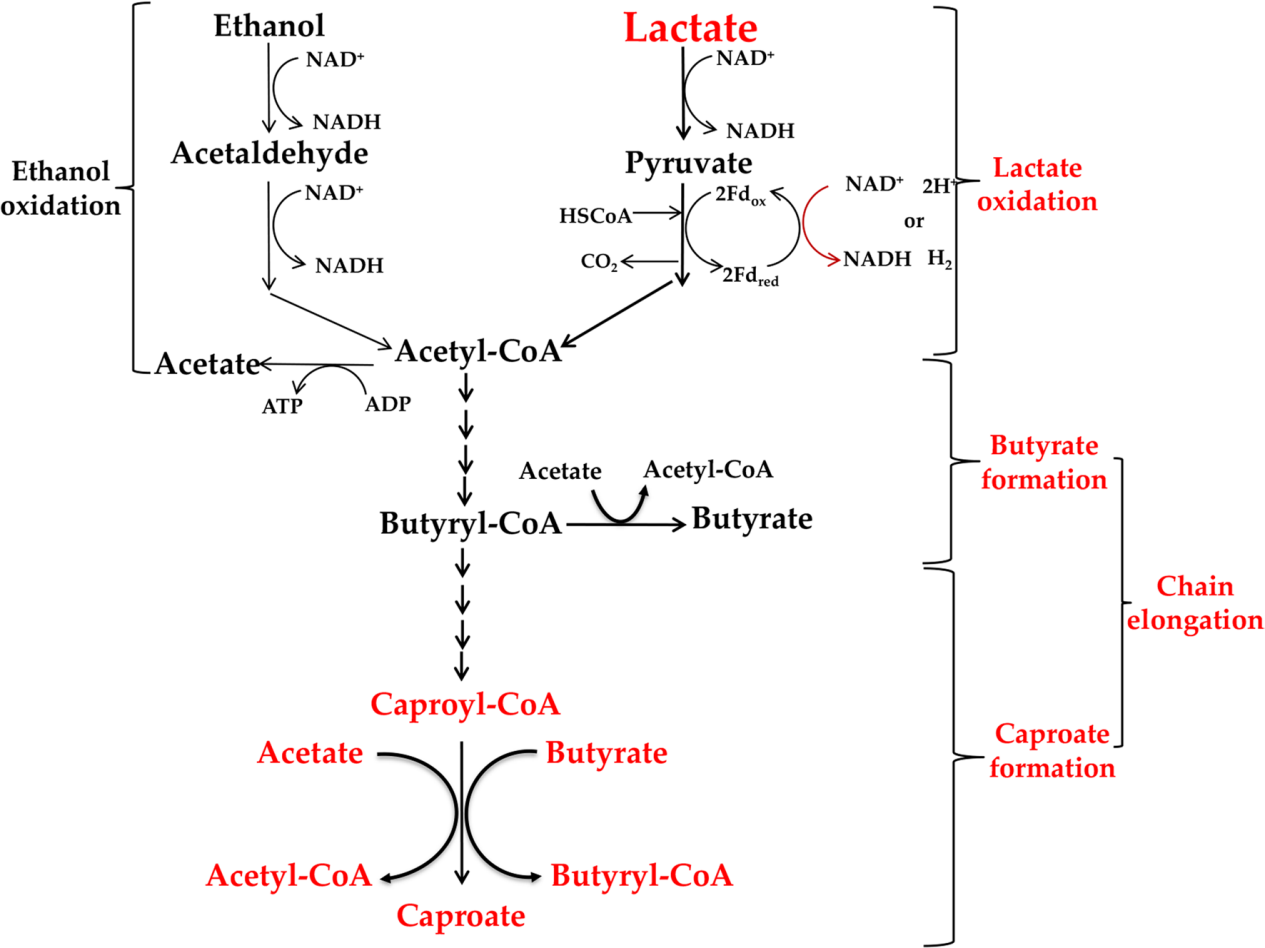
Proposed metabolic pathways for butyrate and caproic acid (CA) formation in strain CPB6. This was extended and modified from previous models for CA production [2, 5] with the combination of lactate oxidation and chain elongation. Pathways were also included here indicating that CA could be formed from condensation of either acetate and caproyl-CoA or butyrate and caproyl-CoA

Based on these results, we propose that the pathway for CA production from lactate in strain CPB6 might be similar to the reverse β-oxidation pathway used for the conversion of ethanol to CA. That is, lactate is converted to acetyl-CoA and this process provides energy to promote the chain elongation (Fig. 7). Although this looks highly logical based on our results from this study, it warrants further investigation through more systematic transcriptomic analysis, isotope-labeled flux analysis, or genetic engineering manipulation. Such attempts are currently underway in our laboratory.

## Conclusion

We reported a newly isolated *Ruminococcaceae* bacterium CPB6, which can use lactate as the feedstock to produce CA as the main product. The CA production capability (final titer, selectivity, and productivity) is much higher than the other reported CA-producing pure cultures. Strain CPB6 prefers acidic initial condition (pH 5.0–6.5) for CA production, and has the optimum growth at the temperature ranging from 30 to 40 °C. High concentration of CA (16.6 g/L) was attained from lactate-containing wastewater by strain CPB6 in fed-batch fermentation under non-sterilized condition, with a maximum CA productivity of 5.29 g/L/day. Both acetate and butyrate are likely involved in the formation of CA from caproyl-CoA, but the activity of caproyl-CoA:acetate CoA transferase is higher than that of caproyl-CoA:butyrate CoA transferase. Lactate is an important intermediate in the anaerobic digestion of wastewaters containing high COD loading from various municipal or industrial processes. Moreover, lactate is also a ubiquitous liquid product easily obtained from lignocellulosic resource and other low-cost feedstock such as corncob molasses. Given the excellent CA production capability of strain CPB6 from non-sterilized lactate-containing wastewater, it can be considered as a promising workhorse for recovering value-added bioproducts from waste streams.

## Methods

### Medium and strain isolation

Strain CPB6 was isolated by using culture plates (15 g/L agar was supplemented besides the fermentation medium, see below) from 10^−3^ to 10^−5^ dilutions of the unique microbiome_ENREF_11 that can produce CA from lactate as we reported [18]. The formation of CA was examined in an anaerobic fermentation medium containing d, l-lactate as the sole energy source at 30 °C. The ingredients in the fermentation medium included (per L): d, l-sodium lactate 4.8 g, KH_2_PO_4_ 1.4 g, (NH_4_)_2_SO_4_ 0.6 g, KCl 1.5 g, MgCl_2_ 0.32 g, CaCl_2_ 0.15 g, selenite–tungstate solution 1.0 mL, yeast extract 1.0 g, l-cysteine 0.25 g, Na_2_S·9H_2_O 0.25 g, and resazurin 0.5 mg. In addition, 1.0 mL of trace element stock solution was also supplemented into the 1 L medium [18]. The pH of the medium was adjusted to 5.5 before sterilization. The fermentation medium was flushed with nitrogen gas for 20 min to remove trace amount of oxygen and then sterilized at 121 °C and 0.1–0.15 MPa for 20 min. After autoclaving, 1.0 mL of vitamin stock solution from an anoxic and filter-sterilized stock solution was added [18]. The same procedure was employed for all following fermentation experiments for oxygen removal and sterilization before inoculation.

### Effects of initial pH and temperature on CA formation from lactate

Batch experiments were performed in 150-mL serum bottles containing 50 mL of fermentation medium. To study the effects of pH on CA production, the pH value of the fermentation medium was adjusted before inoculation to 5.0, 5.5, 6.0, 6.5, 7.0, 7.5, and 8.0, respectively, by adding 2 M sterile HCl or NaOH. The preculture of CPB6 was first cultivated for 3 days in a seed medium containing the following reagents (per L): peptone 1.25 g, yeast extract 1.25 g, dextrose 3.0 g, NaCl 0.08 g, l-cysteine-HCl × H_2_O 0.25 g, KCl 1.5 g, MgSO_4_·7H_2_O 0.008 g, CaCl_2_ 0.008 g, K_2_HPO_4_ 0.08 g, NaHCO_3_ 0.4 g, vitamin K_1_ 0.005 g, and Hemin 0.0125 g (adjusted to pH 5.5 before inoculation). Then, 2.5 mL of the preculture was inoculated into the main fermentation medium with approximately 1.5 g/L of acetate supplemented as electron acceptor, the headspace of which was filled with 100% N_2_. The fermentation culture was incubated at 30 °C in a rotating shaker with a shaking rate of 180 rpm. To investigate the effects of temperature on CA production, the fermentation culture was prepared similarly as described above (but all started with pH 5.5) and incubated at 20, 30, 40, or 50 °C, respectively. Both experiments were performed in triplicate.

### Using real wastewater as carbon source for CA production

The food wastewater used in this study was collected from the fermentation pit of a liquor brewing factory located in Sichuan, China. The main characteristics of this wastewater were pH 3.8 ± 0.2, lactate 116.8 ± 4.2 g/L, acetate 1.5 ± 0.08 g/L, butyrate 1.0 ± 0.02 g/L, glucose 15.6 ± 2.2 g/L, and ethanol 20.5 ± 2.9 g/L. The municipal wastewater used in this study was collected from the influent of a wastewater treatment plant located in Sichuan, China. The main characteristics of this wastewater were TCOD (total chemical oxygen demand) 270–310 mg COD/L, SCOD (soluble chemical oxygen demand) 170–215 mg COD/L, acetate 50–80 mg COD/L, TN (total nitrogen) 26–37 mg/L, and TP (total phosphorus) 3.0–5.5 mg/L, pH 7.4–7.6. The food wastewater and municipal wastewater were mixed at a 1:3 volume ratio. The final pH was adjusted to 5.5 with 2 M HCl and 2 M NaOH. Without oxygen removal and sterilization, the preculture strain CPB6 was inoculated into the mixed wastewater at an inoculum volume ratio of 5%. Meanwhile, as a control, the same fermentation was also conducted using autoclave-sterilized wastewater. The experiment was performed in a 150-mL serum bottle with 50 mL fermentation medium at 30 °C with an agitation rate of 180 rpm. The experiment was performed in triplicate. When the lactate in the fermentation broth was used up (after about three days), fresh mixed wastewater (the autoclaved wastewater was used for the control) was supplemented into the fermentation, making lactate concentration up to approximately 15 g/L. Then the fermentation was kept running for about another three days until the end. The operational conditions (including inoculum ratio, fermentation volume, temperature, agitation rate, triplicate) were all kept the same for the following experiments unless otherwise indicated.

### Effects of acetate and butyrate on CA formation from lactate

In order to investigate the effects of acetate and butyrate on CA production, experiments were carried out with following three different compositions as the main organic carbon sources: 5 g/L lactate (Group 1), 5 g/L lactate + 5 g/L acetate (Group 2), and 5 g/L lactate + 5 g/L butyrate (Group 3).

### Enzymatic assays with crude cell extract

Enzymatic assays with crude cell extract were performed to verify the existence of several key reactions and relative enzymes in the chain elongation pathways in strain CPB6. For each assay, cells of 1.5 mL preculture with an OD_600_ of 0.8 were washed with 1× PBS solution (pH 5.5) for three times, and crude cell extract was prepared from the cells using CelLytic B reagent (Sigma–Aldrich Corporation, St Louis, MO, USA) following the manufacturer’s instruction. The assays were conducted at 35 °C in 1.5-mL sealed centrifuge tubes containing substrates as listed below: caproyl-CoA (Sigma-Aldrich) + acetate, caproyl-CoA + butyrate, and butyryl-CoA (Sigma-Aldrich) + acetate. The final concentration of each substrate was 4.0 mM. Then ATP (Sigma-Aldrich) was added into the reaction with a final concentration of 0.2 mM. Control tests were also conducted at the same time following the same procedure but crude cell extract was replaced with the same amount of 1× PBS solution. All these tests were performed in triplicate in an anaerobic chamber (BactronEZ, Shellab, USA) which was kept under anaerobic condition all the time with mixed gases (CO_2_: H_2_: N_2_ = 5:5:90). Reaction rates for the tests were calculated based on the amount of CA/butyrate generated or the amount of butyrate/acetate consumed within 12 h.

### Analytical methods

Liquid samples were collected regularly from each experiment and centrifuged for 5 min at 10,000 rpm. The supernatants were then diluted fivefold with distilled water and filtrated through 0.22-μm filter (Millipore Corp., Bedford, MA). Carboxylates (C1–C6), lactate, glucose, and ethanol concentrations were determined using an HPLC (Agilent 1260 Infinity, USA) equipped with a differential refraction detector (RID) and a Hi-Plex H column (300 × 6.5 mm) [18] _ENREF_11. Hydrogen, carbon dioxide, and methane were measured using an Agilent 6890 gas chromatography (GC) system (Agilent Technologies, USA) with a thermal conductivity detector (TCD) and a 2-m stainless steel column packed with Porapak Q (50/80 mesh)_ENREF_11 [18].

## Additional files

**Additional file 1.** Materials and methods.

**Additional file 2: Figure S1.** SEM image of Ruminococcaceae bacterium CPB6. **Figure S2.** Effects of pH and temperature on strain CPB6 growth and its influence on caproate production rate at 96 h: growth A (pH) and B (temperature); caproate production rate: C (pH) and D (temperature). **Figure S3.** pH values before and after the reaction. **Figure S4.** Caproic acid (CA) recovery from lactate-containing wastewater by strain CPB6 under sterilized conditions. The fermentation was started using the mixed wastewater as described in the Materials and Methods section. When the wastewater was depleted, additional wastewater was added to support the further CA production. **Figure S5.** Fermentation profiles of strain CPB6 when acetate (A) and butyrate (B) was used as the sole carbon source (without lactate provided).

## Abbreviations

CA: *n*-caproic acid
COD: chemical oxygen demand
SCFAs: short-chain fatty acids
MCFAs: medium-chain fatty acids
HRT: hydraulic retention time
TCOD: total chemical oxygen demand
SCOD: soluble chemical oxygen demand
TN: total nitrogen
TP: total phosphorus
GC: gas chromatography
HPLC: high-performance liquid chromatography

## References

[1] Angenent LT, Richter H, Buckel W, Spirito CM, Steinbusch KJJ, Plugge CM, Strik DPBTB, Grootscholten TIM, Buisman CJN, Hamelers HVM (2016). Chain elongation with reactor microbiomes: open-culture biotechnology to produce biochemicals. Environ Sci Technol.

[2] Spirito CM, Richter H, Rabaey K, Stams AJ, Angenent LT (2014). Chain elongation in anaerobic reactor microbiomes to recover resources from waste. Curr Opin Biotechnol.

[3] Desbois AP (2012). Potential applications of antimicrobial fatty acids in medicine, agriculture and other industries. Recent Patents Anti-Infect Drug Disc..

[4] Barker HA, Kamen MD, Bornstein BT (1945). The synthesis of butyric and caproic acids from ethanol and acetic acid by *Clostridium kluyveri*. Proc Natl Acad Sci.

[5] Seedorf H, Fricke WF, Veith B, Brüggemann H, Liesegang H, Strittmatter A, Miethke M, Buckel W, Hinderberger J, Li F, Hagemeier C, Thauer RK, Gottschalk G (2008). The genome of *Clostridium kluyveri*, a strict anaerobe with unique metabolic features. Proc Natl Acad Sci.

[6] Steinbusch KJJ, Hamelers HVM, Plugge CM, Buisman CJN (2010). Biological formation of caproate and caprylate from acetate: fuel and chemical production from low grade biomass. Energy Environ Sci.

[7] Grootscholten TIM, Steinbusch KJJ, Hamelers HVM (2013). Improving medium chain fatty acid productivity using chain elongation by reducing the hydraulic retention time in an upflow anaerobic filter. Bioresour Technol.

[8] Agler MT, Spirito CM, Usack JG, Werner JJ, Angenent LT (2014). Development of a highly specific and productive process for *n*-caproic acid production: applying lessons from methanogenic microbiomes. Water Sci Technol.

[9] Agler MT, Wrenn BA, Zinder SH, Angenent LT (2011). Waste to bioproduct conversion with undefined mixed cultures: the carboxylate platform. Trends Biotechnol.

[10] Ge S, Usack JG, Spirito CM, Angenent LT (2015). Long-term *n*-caproic acid production from yeast-fermentation beer in an anaerobic bioreactor with continuous product extraction. Environ Sci Technol.

[11] Kleerebezem R, van Loosdrecht MC (2007). Mixed culture biotechnology for bioenergy production. Curr Opin Biotechnol.

[12] Raunkjær K, Hvitved-Jacobsen T, Nielsen PH (1994). Measurement of pools of protein, carbohydrate and lipid in domestic wastewater. Water Res.

[13] Arslan D, Steinbusch K, Diels L, De Wever H, Hamelers H, Buisman C (2013). Selective carboxylate production by controlling hydrogen, carbon dioxide and substrate concentrations in mixed culture fermentation. Bioresour Technol.

[14] Kucek LA, Nguyen M, Angenent LT (2016). Conversion of l-lactate into *n*-caproate by a continuously fed reactor microbiome. Water Res.

[15] Marounek M, Fliegrova K, Bartos S (1989). Metabolism and some characteristics of ruminal strains of *Megasphaera elsdenii*. Appl Environ Microbiol.

[16] Agler MT, Werner JJ, Iten LB, Dekker A, Cotta MA, Dien BS, Angenent LT (2012). Shaping reactor microbiomes to produce the fuel precursor *n*-butyrate from pretreated cellulosic hydrolysates. Environ Sci Technol.

[17] Andersen SJ, Candry P, Basadre T, Khor WC, Roume H, Hernandez-Sanabria E, Coma M, Rabaey K (2015). Electrolytic extraction drives volatile fatty acid chain elongation through lactic acid and replaces chemical pH control in thin stillage fermentation. Biotechnol Biofuels.

[18] Zhu X, Tao Y, Liang C, Li X, Wei N, Zhang W, Zhou Y, Yang Y, Bo T (2015). The synthesis of *n*-caproate from lactate: a new efficient process for medium-chain carboxylates production. Sci Rep..

[19] Counotte GH, Prins RA, Janssen RH, Debie MJ (1981). Role of *Megasphaera elsdenii* in the fermentation of dl-[2-^13^C] lactate in the rumen of dairy cattle. Appl Environ Microbiol.

[20] Sträuber H, Lucas R, Kleinsteuber S (2015). Metabolic and microbial community dynamics during the anaerobic digestion of maize silage in a two-phase process. Appl Microbiol Biotechnol.

[21] Duncan SH, Louis P, Flint HJ (2004). Lactate-utilizing bacteria, isolated from human feces, that produce butyrate as a major fermentation product. Appl Environ Microbiol.

[22] Tao Y, Hu X, Zhu X, Jin H, Xu Z, Tang Q, Li X (2004). Production of butyrate from lactate by a newly isolated *Clostridium* sp. BPY5. Appl Biochem Biotechnol.

[23] Van den Abbeele P, Belzer C, Goossens M, Kleerebezem M, De Vos WM, Thas O, De Weirdt R, Kerckhof F-M, Van de Wiele T (2013). Butyrate-producing *Clostridium* cluster XIVa species specifically colonize mucins in an in vitro gut model. ISME J.

[24] Tsukahara T, Hashizume K, Koyama H, Ushida K (2006). Stimulation of butyrate production through the metabolic interaction among lactic acid bacteria, *Lactobacillus acidophilus*, and lactic acid-utilizing bacteria, *Megasphaera elsdenii*, in porcine cecal digesta. Anim Sci J..

[25] Weimer PJ, Moen GN (2013). Quantitative analysis of growth and volatile fatty acid production by the anaerobic ruminal bacterium *Megasphaera elsdenii* T81. Appl Microbiol Biotechnol.

[26] Cheon Y, Kim J-S, Park J-B, Heo P, Lim J, Jung G, Seo J-H, Park J, Koo H, Cho K, Park J-B, Ha S-J, Kweon D-H (2014). A biosynthetic pathway for hexanoic acid production in *Kluyveromyces marxianus*. J Biotechnol.

[27] Roddick FA, Britz ML (1997). Production of hexanoic acid by free and immobilised cells of *Megasphaera elsdenii*: influence of in situ product removal using ion exchange resin. J Chem Technol Biotechnol.

[28] Choi K, Jeon BS, Kim B-C, Oh M-K, Um Y, Sang B-I (2013). In situ biphasic extractive fermentation for hexanoic acid production from sucrose by *Megasphaera elsdenii* NCIMB 702410. Appl Biochem Biotechnol.

[29] Jeon BS, Kim B-C, Um Y, Sang B-I (2010). Production of hexanoic acid from d-galactitol by a newly isolated *Clostridium* sp. BS-1. Appl Microbiol Biotechnol.

[30] Weimer PJ, Stevenson DM (2011). Isolation, characterization, and quantification of *Clostridium kluyveri* from the bovine rumen. Appl Microbiol Biotechnol.

[31] Yu H-Q, Mu Y (2006). Biological hydrogen production in a UASB reactor with granules. II: reactor performance in 3-year operation. Biotechnol Bioeng.

[32] Dekishima Y, Lan EI, Shen CR, Cho KM, Liao JC (2011). Extending carbon chain length of 1-butanol pathway for 1-hexanol synthesis from glucose by engineered *Escherichia coli*. J Am Chem Soc.

[33] González-Cabaleiro R, Lema JM, Rodríguez J, Kleerebezem R (2013). Linking thermodynamics and kinetics to assess pathway reversibility in anaerobic bioprocesses. Energy Environ Sci.

