# Supplementary material for: Production of high-concentration n-caproic acid from lactate through fermentation using a newly isolated Ruminococcaceae bacterium CPB6
Source: Biotechnol Biofuels. 2017 Apr 21;10:102. doi: 10.1186/s13068-017-0788-y (PMC5399333; doi:10.1186/s13068-017-0788-y)
Supplement: Supplementary file 1 — Additional file 1. Materials and methods. [file 13068_2017_788_MOESM1_ESM.pdf]

Additional file 1

1. MATERIALS AND METHODS

1.1 16S rRNA Sequencing. Genomic DNA was extracted using the TIANamp Bacteria DNA kit (TIANGEN Biotech, China). 16S rRNA sequences were amplified with a universal primer set, 27f (AGAGTTTGATCMTGGCTCAG) and 1492r (ACGGCTACCTTGTTACGACTT). PCR amplification system (50 μl) includs: buffer (10×) 5 μl, $MgCl_2$ (25 mM) 4 μl, dNTP (10 mM) 1 μl, primer 27f (10 μM) 1 μl, primer 1492r (10 μM) 1 μl, Taq enzyme (5 U/μl) 0.5 μl, template DNA 1.0 μl, and sterile water 36.5μl. PCR amplification was performed following the program as below: pre-denaturation at 94 ^{o}C for 5 min; then 30 cycles were performed with denaturation at 94 ^{o}C for 1 min, annealing at 55 ^{o}C for 1 min, 72 °C extension for 2 min;, a final extension at 72 ^{o}C was performed for 10 min. The PCR product was purified using the TIANgel Midi Purification kit (TIANGEN Biotech, China), cloned to the pM18-T (TaKaRa) and sequenced using an automated DNA sequencer (model ABI3730; Applied BioSystems). Similarity of the 16S rRNA sequences from the isolates to those from other isolated organisms was compared using the Ezbiocloud (http://www.ezbiocloud.net/), GenBank, and RDP (http://rdp.cme.msu.edu/), respectively.

1.2 Scanning Electron Microscopy (SEM). The culture of strain CPB6 was centrifuged at 1000 rpm for 5 min, and the pellet was washed 3 times with 0.1 M phosphate buffer (pH 7.4), and fixed in 0.1 M phosphate buffer (pH 7.4) containing 2.5% glutaraldehyde at 4 ºC for 4 h. After rinsing twice with 0.1 M phosphate buffer

(pH 7.4), the samples were dehydrated in ethanol serials (50%, 70%, 80%, 90%, and 100%, 15 min per step), and then dried in air. Finally, the images were obtained using the FEI Quanta 200 SEM at 20 kV.
