# Supplementary material for: Production of high-concentration n-caproic acid from lactate through fermentation using a newly isolated Ruminococcaceae bacterium CPB6
Source: Biotechnol Biofuels. 2017 Apr 21;10:102. doi: 10.1186/s13068-017-0788-y (PMC5399333; doi:10.1186/s13068-017-0788-y)
Supplement: Supplementary file 2 — Additional file 2: Figure S1. SEM image of Ruminococcaceae bacterium CPB6. Figure S2. Effects of pH and temperature on strain CPB6 growth and its influence on caproate production rate at 96 h: growth A (pH) and B (temperature); caproate production rate: C (pH) and D (temperature). Figure S3. pH values before and after the reaction. Figure S4. Caproic acid (CA) recovery from lactate-containing wastewater by strain CPB6 under sterilized conditions. The fermentation was started using the mixed wastewater as described in the Materials and Methods section. When the wastewater was depleted, additional wastewater was added to support the further CA production. Figure S5. Fermentation profiles of strain CPB6 when acetate (A) and butyrate (B) was used as the sole carbon source (without lactate provided). [file 13068_2017_788_MOESM2_ESM.pdf]

# Additional file 2

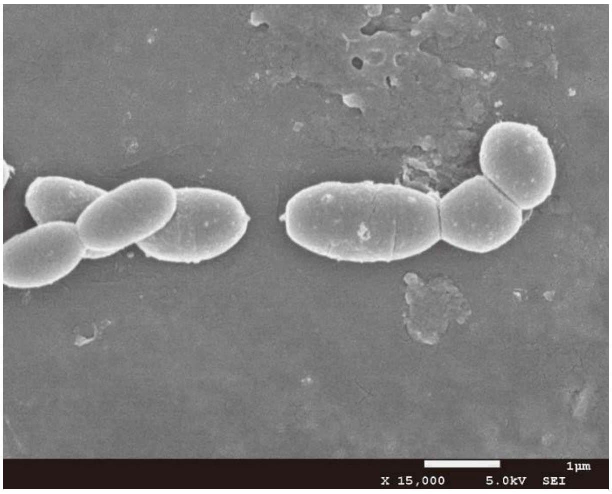


Figure S1 SEM image of *Ruminococcaceae* bacterium CPB6.

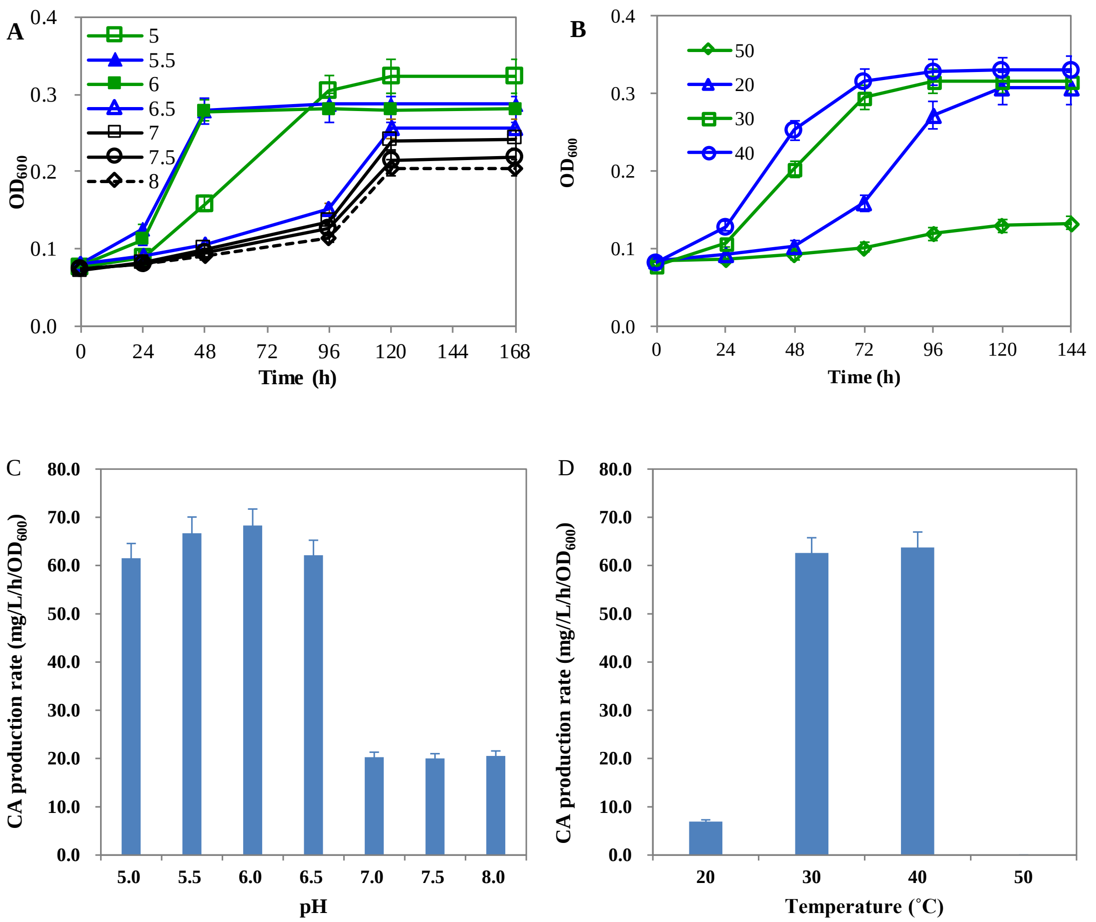


Figure S2 Effects of pH and temperature on strain CPB6 growth and its influence on caproate production rate at 96 h: growth A (pH) and B (temperature); caproate production rate: C (pH) and D (temperature)

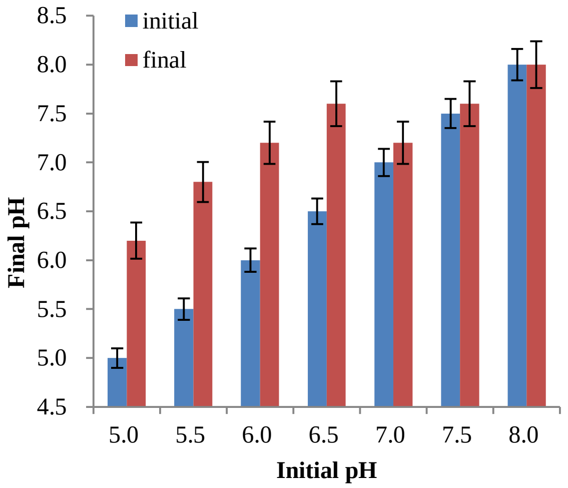


Figure S3. pH values before and after the reaction.

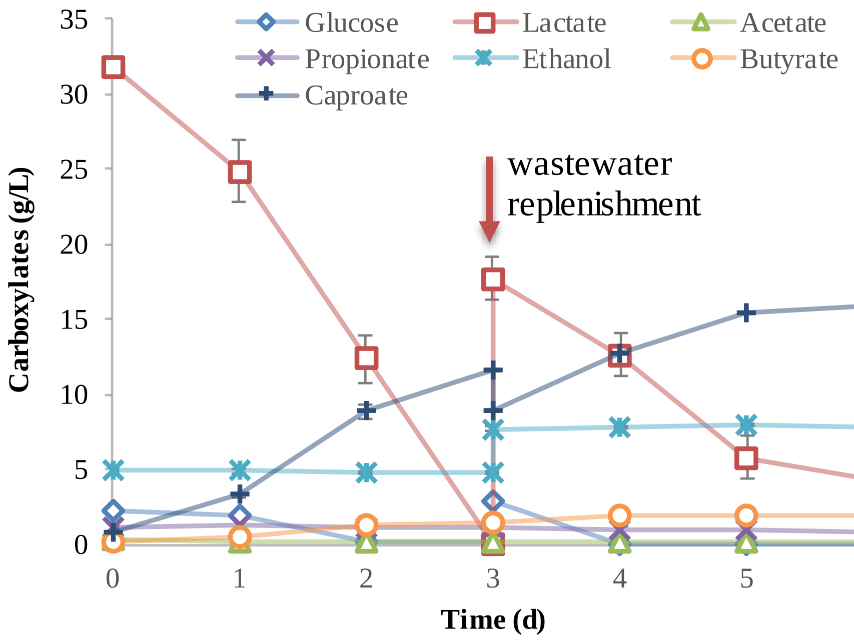


Figure S4 Caproic acid (CA) recovery from lactate-containing wastewater by strain CPB6 under sterilized conditions. The fermentation was started using the mixed wastewater as described in the Materials and Methods section. When the wastewater was depleted, additional wastewater was added to support the further CA production.

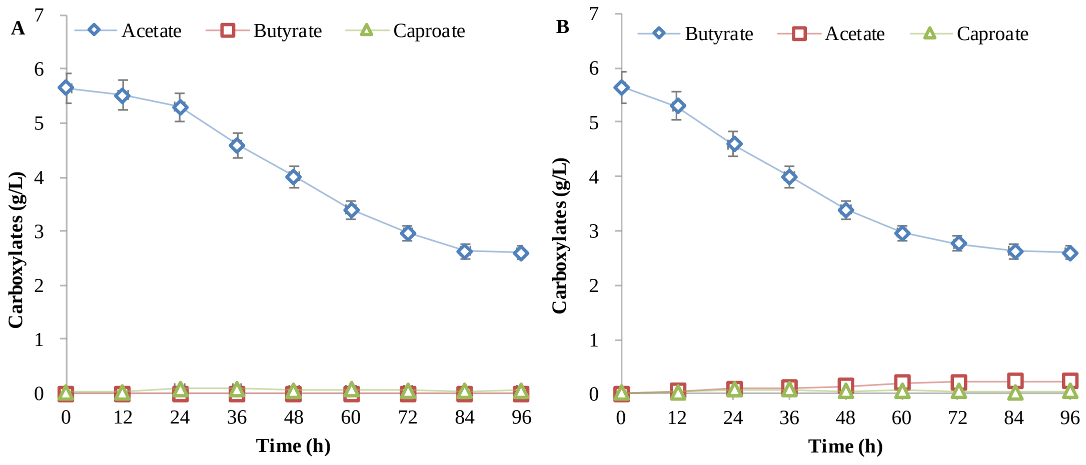


Figure S5 Fermentation profiles of strain CPB6 when acetate (A) and butyrate (B) was used as the sole carbon source (without lactate provided).
